# A prospective multicenter validation study of a machine learning algorithm classifier on quantitative electroencephalogram for differentiating between dementia with Lewy bodies and Alzheimer’s dementia

**DOI:** 10.1371/journal.pone.0265484

**Published:** 2022-03-31

**Authors:** Yukiko Suzuki, Maki Suzuki, Kazue Shigenobu, Kazuhiro Shinosaki, Yasunori Aoki, Hirokazu Kikuchi, Toru Baba, Mamoru Hashimoto, Toshihiko Araki, Kristinn Johnsen, Manabu Ikeda, Etsuro Mori

**Affiliations:** 1 Department of Behavioral Neurology and Neuropsychiatry, United Graduate School of Child Development, Osaka University, Suita, Osaka, Japan; 2 Department of Psychiatry, Graduate School of Medicine, Osaka University, Suita, Osaka, Japan; 3 Department of Psychiatry, Asakayama General Hospital, Sakai, Osaka, Japan; 4 Department of Psychiatry, Nippon Life Hospital, Osaka, Osaka, Japan; 5 Division of Neurology, Tohoku Medical and Pharmaceutical University, Sendai, Miyagi, Japan; 6 Department of Neurology, National Hospital Organization Sendai Nishitaga Hospital, Sendai, Miyagi, Japan; 7 Department of Neuropsychiatry, Faculty of Medicine, Kindai University, Osakasayama, Osaka, Japan; 8 Division of Medical Technology, Osaka University Hospital, Suita, Osaka, Japan; 9 Research and Development, Mentis Cura, Reykjavík, Iceland; Utano National Hospital, JAPAN

## Abstract

**Background and purpose:**

An early and accurate diagnosis of Dementia with Lewy bodies (DLB) is critical because treatments and prognosis of DLB are different from Alzheimer’s disease (AD). This study was carried out in Japan to validate an Electroencephalography (EEG)-derived machine learning algorithm for discriminating DLB from AD which developed based on a database of EEG records from two different European countries.

**Methods:**

In a prospective multicenter study, patients with probable DLB or with probable AD were enrolled in a 1:1 ratio. A continuous EEG segment of 150 seconds was recorded, and the EEG data was processed using MC-004, the EEG-based machine learning algorithm, with all clinical information blinded except for age and gender.

**Results:**

Eighteen patients with probable DLB and 21 patients with probable AD were the included for the analysis. The performance of MC-004 differentiating probable DLB from probable AD was 72.2% (95% CI 46.5–90.3%) for sensitivity, 85.7% (63.7–97.0%) for specificity, and 79.5% (63.5–90.7%) for accuracy. When limiting to subjects taking ≤5 mg donepezil, the sensitivity was 83.3% (95% CI 51.6–97.9), the specificity 89.5% (66.9–98.7), and the accuracy 87.1% (70.2–96.4).

**Conclusions:**

MC-004, the EEG-based machine learning algorithm, was able to discriminate between DLB and AD with fairly high accuracy. MC-004 is a promising biomarker for DLB, and has the potential to improve the detection of DLB in a diagnostic process.

## Introduction

Dementia with Lewy bodies (DLB) and Alzheimer’s disease (AD) are the most common causes of neurodegenerative dementia in the elderly, and can be of a similar presentation, with an overlap of clinical symptoms. An early and accurate diagnosis of DLB is critical because it can lead to early initiation of effective treatment for cognitive and psychiatric disorders, such as acetylcholinesterase inhibitors (AChEIs), and avoidance of potentially life-threatening treatments such as antipsychotic drugs, which are known to increase the risk of serious side effects in patients with DLB [1, 2]. Additionally, earlier and more accurate diagnosis of DLB is expected to reduce burdens on patients and caregivers and be of social benefit in reducing the costs of nursing and medical care.

The diagnosis of DLB relies on a series of consensus criteria based on symptomatic features and supplementary tests that were first described in 1996 and subsequently revised in 2005 and 2017 [3–5]. The first version of these clinical diagnostic criteria had a limitation in terms of poor sensitivity, albeit high specificity, when compared with neuropathological findings [6, 7]. To overcome this limitation, the latest version of the consensus criteria includes features typical of Lewy body-type pathology as “indicative” biomarkers of DLB. These are reduced uptake of dopamine transporter in the basal ganglia as measured by ^123^I-ioflupane single-photon emission tomography (DaTSCAN^®^), low uptake of iodine-123 metaiodobenzylguanidine (^123^I-MIBG) in the myocardium as measured by ^123^I-MIBG myocardial scintigraphy (MIBG scintigraphy), and REM sleep without atonia as detected by polysomnography. The inclusion of indicative biomarkers in the latest version of the DLB consensus criteria may improve the differential diagnosis between DLB and AD. Nevertheless, it is still problematic to differentiate between DLB and AD. These biomarkers are underutilized in clinical practice, and DLB tends to be misdiagnosed as AD. In addition, all three biomarkers require expensive procedures and the number of facilities where they can be used is limited. In clinical settings, up to 80% of DLB patients are misdiagnosed as AD [8].

Electroencephalography (EEG) is a low-cost, non-invasive and widely available tool that gives a functional measure of neuronal and synaptic integrity. Numerous studies have examined the diagnostic value of EEG as an additional diagnostic investigation for DLB. The role of EEG in diagnosing DLB and differentiating it from other neurodegenerative diseases has recently been discussed in a systematic review [9]. According to it, EEG may be efficient as a diagnostic tool in DLB. Studies of visual analysis of EEG have identified characteristics to DLB including generalized slowing of the EEG, temporal slow wave transients, frontal intermittent delta activity, a slowing of the dominant alpha frequency range named as “pre-alpha” rhythm at occipital regions as hallmarks of DLB, while AD is associated with slowing in the temporal areas. An attempt has been made to correlate neurological/physiological characteristics with EEG patterns for DLB and AD [10]. Quantitative EEG (qEEG) analysis has been applied to overcome the inherent limitations of visual EEG analysis which includes the inter-rater variability along with the low sensitivity in recognizing subtle abnormalities, providing a way to differentiate subjects with dementia due to DLB from those with AD. The aforementioned systematic review noted that as compared with AD, DLB is associated with a greater fluctuation in mean EEG frequency, a sparse appearance of alpha rhythm, a greater dominant frequency variability, a higher degree of overall coherence in the delta and theta bands, a lower degree of overall coherence in the alpha band, and compromise of functional cortical connectivity [9]. The 2017 consensus criteria incorporated prominent posterior slow wave EEG activity with periodic fluctuations in the prealpha/theta range as a supportive diagnostic biomarker. However, published data are insufficient to categorize implementation of EEG features as an indicative biomarker in the differential diagnosis of DLB and AD. This is because no standards have been applied for EEG reading and interpretation, thresholds for test positivity have not been defined objectively for EEG, high level evidence including data from prospective, multicenter, validation studies is lacking, and data correlating antemortem EEG features with postmortem pathology is absent. There is an urgent need for applying a standardization for the EEG analysis procedures and replicating the diagnostic properties in large cohorts [9].

In recent years, it has been reported that the analysis of qEEG records using machine learning enables the differential diagnosis of dementia, including DLB and AD [11–13]. The study by Snaedal et al. [13] in Iceland reported that the SPR (statistical pattern recognition) method was useful to differenciate cases of degenerative disorders. In a single center study, Garn et al. [11] reported a 100% classification accuracy for AD vs. DLB+ Parkinson’s disease dementia (PDD) in leave-one-out cross-validation using 25 QEEG features as input to a SPR approach. Both studies reported cross-validation estimates of the properties of the resulting algorithm, in particular the latter was prone to overfitting as the number of features was similar size as the training group. Therefore, the level of clinical evidence is not sufficiently high to warrant clinical application. Engedal et al. [12] conducted a multicenter study in Norway using an qEEG analysis algorithm trained based on the dataset obtained in Iceland [12], in which favorable classifying performance (sensitivity = 84% and specificity = 81%) in differentiating between AD and DLB was demonstrated. Although they adopted a separate test dataset, training and test datasets were from neighboring countries with close ethnicity and culture, which could lead to overfitting and limit generalizability to a different clinical population.

The objective of the present study was to investigate how accurately a machine learning algorithm based on qEEG could classify subjects as probable DLB and probable AD in a country with different ethnicity and culture than the previous study [12]. We used MC-004, a successor of the EEG-based machine learning algorithm classifier used in the study of Engedal et al. [12], which was developed incorporating more optimized technics and training on a larger population. In this prospective, multicenter study, we validated the algorithm developed based on European cohorts in a totally independent cohort, a Japanese patient population, to explore the feasibility of applying it in a pivotal trial.

## Materials and methods

### Subjects

Between April and December 2019, we performed a multicenter study in 5 Japanese sites. Patients with probable DLB or with probable AD were enrolled in a 1:1 ratio. The inclusion criteria were: (1) 50–90 years of age; (2) patients with probable DLB according to the revised consensus criteria [5], or patients with probable AD according to the NIA-AA criteria [14]. Patients who presented only one core DLB feature or only positive indicative DLB biomarkers but otherwise fulfilled the criteria for probable AD (namely classified into both possible AD and possible DLB), and Parkinson’s disease dementia (PDD; i.e., subjects with a diagnosis of Parkinson’s disease at least 1 year prior to onset of dementia) were not included; (3) a mini-mental state examination (MMSE) score of 14–26; (4) patients or responsible caregiver provides informed consent for the subject to participate in the study. We excluded patients who met at least one of the following exclusion criteria to avoid the presence of mixed pathology, ambiguous diagnosis, and influence of drugs on EEG.: (1) patients with vascular lesions likely contributed to the subject’s dementia on MRI or CT; (2) patients with significant neurologic disease other than probable DLB or AD; (3) patients with history of alcohol or drug abuse within the past 2 years; (4) patients with history of schizophrenia; (5) patients with any significant systemic illness or unstable medical condition that may affect EEG; (6) patients using specific medications that possibly influence EEG. AChEIs and levodopa/DOPA decarboxylase inhibitor (DDI) were permitted, while memantine and anti-Parkinsonism medications other than levodopa/DDI were not allowed.

Clinical information was collected using an electronic case report form developed and administered by Osaka University Hospital Data Coordinating Center. The following information were collected: age, gender, years of education, and doses of AChEI and levodopa/DDI. Results of the following tests and assessments were recorded: MMSE [15]; Neuropsychiatric Inventory [16]; Cognitive Fluctuation Inventory [17]; REM sleep Behavior Disorder Screening Questionnaire [18]; Movement Disorder Society-Unified Parkinson’s Disease Rating Scale Part III [19]; Noise Pareidolia Test [20]; Clinical Dementia Rating; head MRI; cerebral blood flow on SPECT; MIBG scintigraphy; and DaTSCAN^®^ imaging. Eligibility was judged by an agreement of two experts in the field of dementia, reviewing full details of all clinical information, neuropsychiatric/neuropsychological assessments, and imaging data.

The study was performed in accordance with the current revision of the Declaration of Helsinki and applicable national and local laws and regulations. All patients gave written informed consent. This study was approved by the Ethical Review Boards of Osaka University and by institutional review boards of all participating centers. This study was registered in the University Hospital Medical Information Network Clinical Trials Registry (UMIN000035926). Recruitment and enrollment began on April 24, 2019 and was completed on December 6, 2019.

### EEG data acquisition

The EEG recording was performed using Nihon Kohden Neurofax EEG systems at each site according to a common harmonized protocol and manual by an experienced technician who had been trained to recognize and eliminate artefacts. The IS 10–20 system was used for electrode placement. The following 19 electrodes were used for the analysis: Fp1, Fp2, F3, F4, F7, F8, Fz, T3, T4, T5, T6, C3, C4, Cz, P3, P4, Pz, O1, and O2. The average potential was used as a reference. Two bipolar electro-oculography channels and one electrocardiogram lead were applied to monitor artefacts. To reduce muscle artefacts in the EEG recording, the patient was comfortable during the recording, sitting in an upright position to encourage to stay fully awake. If the patient needed to be lying down, the technician carefully monitored the patient’s state of alertness. An impedance of 10 kOhms or less in each electrode channel was required, as high impedance influences the extracted qEEG features and may result in an erroneous result. The EEG was recorded for ≥150 seconds during which the subjects were at rest with their eyes closed. The subjects were alerted if they became visibly drowsy. To ensure an acceptable 150-second segment, the EEG technicians were instructed to repeat two or more 5-minute EEG recordings at 1~2-minute intervals and select the 150-second recording that was free of artifacts. The analysis by the MC-004 requires a data segment (epoch) of at least 150 seconds duration comprising clean, eyes closed resting state EEG data. After all personal health information was removed and the data was uploaded to a server set up in Japan via internet.

### EEG analysis by MC-004

The uploaded EEG was processed at Mentis Cura, Oslo, Norway, using MC-004, with the identity of the EEG technician and all clinical information except for age and gender blinded. MC-004 returns a single number as output, which was determined by applying a linear support vector machine learning technique trained on the Mentis Cura DLB/PDD and AD dataset. The index is scaled based on the training dataset, such that the optimal cut-off value for the index is 0, and the magnitude scale is given in terms of the standard deviation of the AD training cohort index values. If the index is below the pre-defined threshold of 0, the individual is likely to have AD. Conversely, if the index is equal to or greater than the pre-defined threshold of 0 the individual is likely to have DLB.

The background and technical features of the MC-004 are briefly described below. The technical aspects of the algorithm building have been published more in detail elsewhere, albeit addressing another research question [21]. MC-004 algorithm was established by improving the algorithm described previously [13] and preliminarily tested in the study by Engedal et al. [12]. The improvement entails a different way of extracting the features from the recordings. In the previous algorithm, only 37 far coherences were considered, but in the improved algorithm all possible pairs of electrodes are considered resulting 171 pairs. That results in higher cortical resolution of the electrophysiology considered, yielding an increased potential of differentiation between DLB and AD. Because MC-004 is much more sophisticated than the previous algorithm by using more DLB data in algorithm designing, MC-004 has a potential to perform even better than the previous algorithm.

The Mentis Cura DLB/ PDD and AD dataset, which the algorithm designing was based on, consisted of two cohorts; Cohort 1, 270 patients with probable AD and 51 patients with probable DLB/PDD recruited in the Memory Clinic of the Geriatric Department, National University Hospital, Reykjavik, Iceland between 2005 and 2011; and Cohort 2, 36 patients with probable AD and 64 patients with probable DLB recruited in the Memory Clinic and Movement Disorder Center, Neurology Clinic of the University G. d’Annunzio of Chieti-Pescara, Chieti-Pescara, Italy from 2009 to 2018. Each patient’s diagnosis was made according to NINCDS-ADRDA criteria [22] and the consensus criteria of McKeith et al. [4] on the basis of all available clinical information including cranial CT/MRI, neuropsychology, and CBF SPECT. In most of the cases of suspected DLB/PDD, DaTSCAN^®^ was performed. There were 115 patients with DLB/PDD (age = 76.3±6.6, men = 58.2%, MMSE = 21.2±5.3) and 306 patients with AD (age = 77.5±7.9, men = 61.5%, MMSE = 22.7±5.3).

The EEG recording was analyzed in 2 sec segments overlapping by 1 sec. The analysis of each segment constituted evaluating all the features the classification relies on, resulting 149 segments analyzed from 150 sec recording. Then, the value of each of the features was estimated applying robust estimates. As this procedure ensured that the estimate was not significantly biased by artefacts due to blinking muscle movement, EKG etc, it can be regarded as automatic artefact removal of the signal. The EEG recordings were pre-processed prior to feature extraction by applying an 8^th^ order Butterworth band-pass filter with the band 0.1–70 Hz. The spectral properties were considered using a ½ Hz resolution. The range used was from 1/2Hz-45Hz leading to 90 complex spectral values for the Fourier transform of each of the 19 (electrodes) +18*19/2 (inter-channel) = 190 timeseries considered, leading to 90*2*190 = 34,200 features in total, as initial input for the development of the algorithm, including 3,420 spectral features and an (18*19/2) *90*2 = 30,780 inter-channel covariance-related features. By comparing the features of all related to the spectral properties from the 2 diagnostic groups, a classifier was trained. Discrete fast Fourier transform was applied to estimate the spectral properties of the signal. If the FFT components for each of the electrodes, segments, and discrete frequencies considered are denoted by *σ*_*cij*_, where *c*∈{1,2,…,19} indicates the channel, *i*∈{1,…,*N*} the segment of the *N* segments considered, and *j*∈{1,…,90} the discrete frequencies $\left(\frac{1}{2}Hz,1Hz,\ldots ,45Hz\right)$, the full spectral resolution covariance between channels *c* and *k* is then expressed by $\chi_{ck}^{ij}{=\sigma }_{cij}*\sigma_{kij}^{*}$. These covariances constitute the base features used for analysis and evaluation of the classification index values. To determine the core features relied on, principal component analysis was applied to each electrode and each covariance. All principal components were then ranked according to their individual discriminatory properties in separating the groups of AD and DLB subjects. The discriminatory properties were determined by evaluating the area under the curve of the receiver operator characteristic based on the training set described above. The two most relevant components from each channel and channel pair were selected for further analysis, leading to 380 distinct features used. If *P*_*ckaj*_ denotes the 2 chosen PCs, *α*∈{1,2}, for electrode pair (*c*,*k*) at frequencies *j*∈{1,…,90}, the core features considered for analysis then become $C_{ck\alpha }=E_{i}\{\sum_{j=1}^{90}{\chi_{ck}^{ij}P}_{ck\alpha j}\}$. The index value for an individual recording is evaluated from these features by $I=\sum_{ck\alpha }C_{ck\alpha }\beta_{ck\alpha }+\beta_{1}^{A}A+\beta_{2}^{A}A^{2}+\rho$, where *A* is the age of the subject in years. The classification coefficients $\beta_{ck\alpha },\;\beta_{i}^{A}$ and *ρ* were determined by using a combination of genetic algorithms to optimize the number of features used, and support vector machine (SVM), a statistical pattern recognition (SPR) technique, was applied to discriminate the AD and DLB groups. This was done separately for men and women, resulting in separate gender dependent indices.

### Statistical analysis

All statistical analyses were performed with SPSS statistics, v.26 (IBM corp.). For binomially distributed data, we assessed differences among the different diagnostic cohorts (probable DLB, probable AD) with respect to patients’ characteristics by means of χ^2^ tests. We used t-tests for normally distributed data; if normality could not be established, we used Mann-Whitney U tests. The primary endpoint of this study was the accuracy of the MC-004 in identifying subjects with probable DLB and subjects with probable AD. The following performance indicators were calculated: sensitivity-the percentage of times that the MC-004 classification was DLB given that the clinical diagnosis was probable DLB; specificity- the percentage of times that the MC-004 classification was AD given that the clinical diagnosis was probable AD; accuracy- the percentage of times the MC-004 diagnosis matched the clinical diagnosis. This was an exploratory, feasibility study in a total of 40 patients. With this sample size, a diagnostic accuracy of more than 65% (based on an earlier study [12]) could be detected with 85% power applying a one-sided α = 5%.

## Results

**Fig 1:**
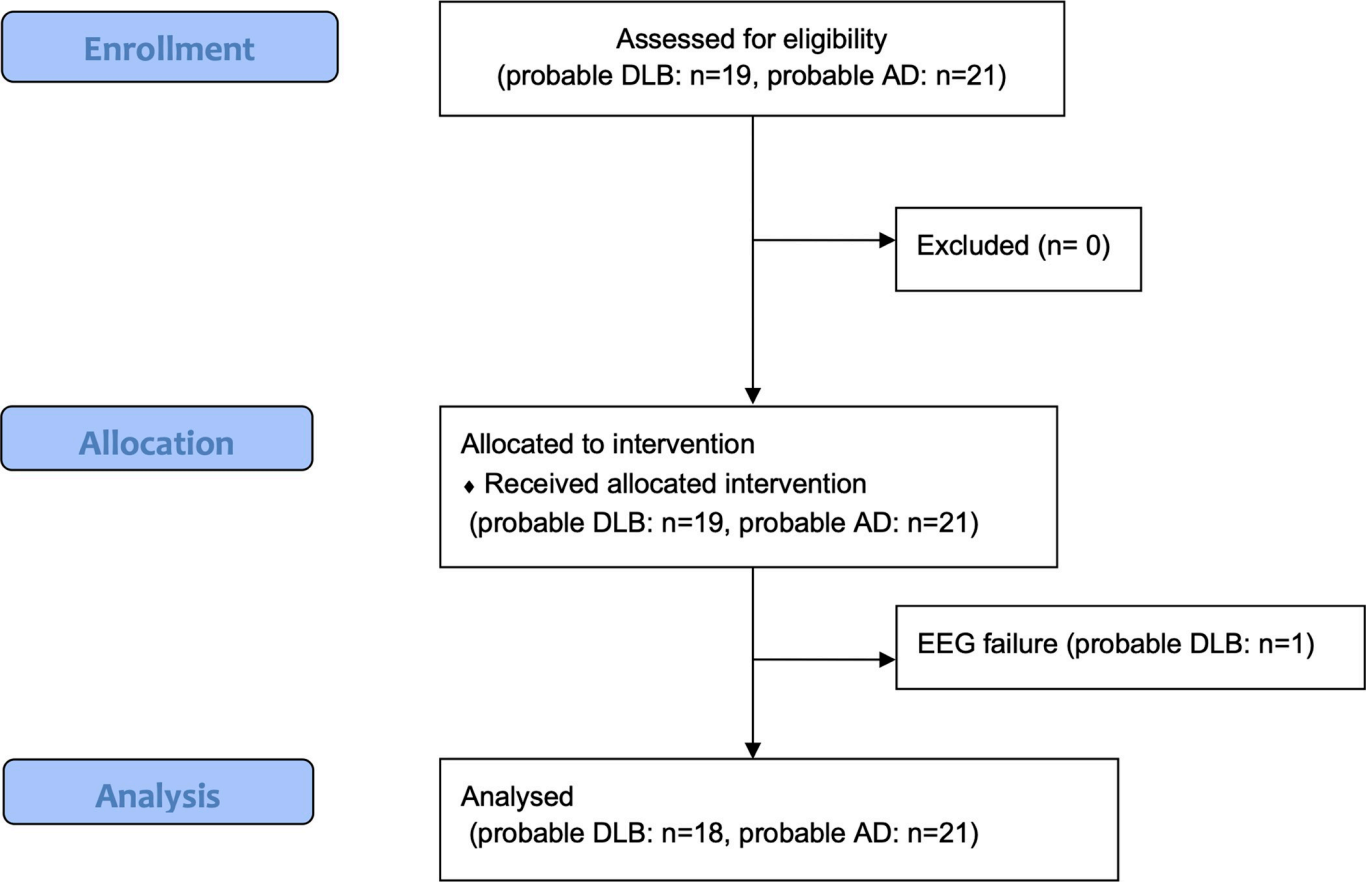
Participant flow chart.

Of the 40 individuals who were enrolled and completed the EEG recording, one subject whose EEG included too much electromyogram-related artefact for analysis was excluded (**Fig 1**). Patients’ characteristics are shown in Table 1. The clinical diagnosis of 18 patients was probable DLB (age = 76.6±5.8, men = 50.0%, MMSE = 21.9±3.1) and that of 21 was probable AD (age = 76.8±6.7, men = 38.8%, MMSE = 21.2±2.9). All patients in the probable DLB group received MIBG scintigraphy (n = 5), DaTSCAN^®^ (n = 7), or both (n = 6), results of which were supportive of DLB except for 1 patient whose DaTSCAN^®^ was not supportive of DLB. Only 1 patient in the AD cohort received DaTSCAN^®^, which was not supportive of DLB.

**Table 1:** Clinical characteristics of the subjects.

	Probable DLB (n = 18)	Probable AD (n = 21)	p value
Sex (M/F)	9/9	8/13	0.672^†^
Age (y)	76.6 (5.8)	76.8 (6.7)	0.79^‡^
Years of education	12.2 (2.8)	12.6 (3.0)	0.67^‡^
MMSE	21.9 (3.1)	21.2 (2.9)	0.52^‡^
CDR (0.5, 1, 2, N/A)	7, 10, 0, 1	6, 12, 2, 1	0.39^‡^
Hoehn & Yahr (0, 1, 2, 3)	1, 3, 8, 6	21, 0, 0, 0	0.000^‡^*
pareidolia test	8.76 (8.2)	0.05 (0.22)	< 0.0000^‡^*
NPI_12	11.6(12.4)	5.2(4.3)	0.0335^‡^*
NPI-visual hallucination	2.53(2.7)	0.0	< 0.0000^‡^*
CFI	2.35(2.396)	0.0	< 0.0000^‡^*
123I-MIBG scan(positive/negative)	11(11/0)	0	-
DaTSCAN^®^ (positive/negative)	13(12/1)	1(0/1)	-
Patients taking AchEI			
donepezil (≦5mg, >5mg)	13(7, 6)	11(9, 2)	-
other than donepezil (more than half the maximum doses in Japan)^a^	0	5 (5)	-
Patients taking levodopa/DDI	5	0	0.015^†^*

Date is mean (SD).

^†^χ2 test

^‡^Mann-Whitney U test

a The maximum doses approved in Japan are 18mg/day for rivastigmine and 24mg/day for galantamine.

MMSE: mini-mental state examination, CDR: clinical dementia rating scale

NPI: neuropsychiatric inventory, CFI: cognitive fluctuation inventory.

Table 2 shows sensitivity and specificity of MC-004 differentiating probable DLB from probable AD. The performance was 72.2% (95% CI 46.5–90.3%) for sensitivity, 85.7% (63.7–97.0%) for specificity, and 79.5% (63.5–90.7%) for accuracy. In ROC (receiver operating characteristic) analysis, AUC (area under the curve) was 0.88. There were 5 subjects in the DLB cohort and 3 subjects in the AD cohort whose MC-004 classification disagreed with the clinical diagnosis. Three of 5 misclassified subjects in the DLB cohort were taking >5 mg of donepezil daily, whereas 3 of 13 correctly classified subjects were taking >5 mg (p = 0.268, Fisher exact probability test). The diagnostic performance of MC-004 was examined when excluding those taking >5 mg donepezil (n = 12 for DLB, n = 19 for AD). The sensitivity was 83.3% (95% CI 51.6–97.9), the specificity 89.5% (66.9–98.7), and the accuracy 87.1% (70.2–96.4). Moreover, when limiting those taking any cholinesterase inhibitors at less than half of the maximum approved doses in Japan (n = 12 for DLB, n = 15 for AD), the sensitivity was 83.3% (95% CI 51.6–97.9), the specificity 86.7% (59.5–98.3), and the accuracy 85.2% (66.3–95.8). On the other hand, 2 of 3 misclassified subjects in the AD cohort did not take any cholinesterase inhibitors.

**Table 2:** Sensitivity and specificity of DLB-index in differentiating between probable DLB and probable AD.

	All subjects	Subjects excluding those taking donepezil > 5mg	Subjects excluding those taking any AChEIs more than half the maximum doses in Japan
n(DLB, AD)	39 (18, 21)	31 (12, 19)	27 (12, 15)
Sensitivity (95% CI)	72.2 (46.5–90.3)	83.3 (51.6–97.9)	83.3 (51.6–97.9)
Specificity (95% CI)	85.7 (63.7–97.0)	89.5 (66.9–98.7)	86.7 (59.5–98.3)
Accuracy (95% CI)	79.5 (63.5–90.7)	87.1 (70.2–96.4)	85.2 (66.3–95.8)

AChEIs: acetylcholinesterase inhibitors.

## Discussion

This prospective, multicenter study carried out in Japan validated an EEG-derived machine learning algorithm for discriminating DLB from AD which developed based on a database of EEG records from two different European countries (Iceland, Italy). Analyzable EEG records were obtained in 97.5% of the patients with mild to moderate dementia attributable to DLB or AD. The overall diagnostic accuracy for differentiating probable DLB from probable AD was 79.5%, with 72.2% for sensitivity and 85.7% for specificity. When excluding those taking >5mg donepezil, the classification performance became somewhat better (sensitivity = 83.3% and specificity = 89.5%). These results suggest that MC-004 is a potentially good tool to discriminate DLB from AD. Further, as the algorithm was validated interracially and cross-culturally, the findings should be broadly applicable.

This diagnostic performance of the algorithm is comparable to that of MIBG scintigraphy or DaTSCAN^®^. In a multicenter, prospective, cross-sectional study, the sensitivity of MIBG scintigraphy was 68.9% and the specificity was 89.1% to differentiate probable DLB from probable AD in both early and delayed images [23]. The three-year follow-up study of the same cohort reported a sensitivity of 77% and a specificity of 97% [24]. In the differential diagnosis between DLB and non-DLB dementia that was predominantly caused by AD, the pivotal study of DaTSCAN^®^ reported a sensitivity of 77.7% and a specificity of 90.4%, and a diagnostic accuracy of 85.7% [25].

The main limitation of this study is the small sample size, which was determined from feasibility considerations rather than a formal sample size calculation. Therefore, the findings should be confirmed in future larger-scale trials. The criterion-related validity is assessed against a gold standard. In this study, the gold standard was not neuropathologic findings but clinical diagnosis according to the latest version of the clinical diagnostic criteria. Although MIBG scintigraphy and DaTSCAN^®^ can enhance the accuracy of the clinical diagnosis of DLB and AD, positivity for either biomarker was not prerequisite for inclusion in the DLB cohort, and negativity for both biomarkers was not used to negate involvement of Lewy body pathology in the AD cohort. As such, uncertainty in the gold standard may have impacted outcomes. The diagnosis of one subject in the DLB cohort whose DaTSCAN^®^ did not support the diagnosis of DLB was questionable. As the real diagnosis of this patient is likely to be AD, although MIBG scintigraphy had not been done, eliminating this patient from the DLB cohort improves sensitivity somewhat. On the other hand, amyloid imaging was not performed, and cerebrospinal fluid-based AD biomarkers were not measured. However, even if these indicate the presence of AD pathology, positivity does not rule out the presence of Lewy body pathology. Up to 50%-80% of patients with DLB have coexisting AD pathology, i.e., amyloid plaques and neurofibrillary tangles [26, 27]. DLB patients with concomitant AD pathology represent a specific diagnostic challenge, as abnormal cerebrospinal fluid AD biomarkers and positive amyloid positron emission tomography [28] can lead to an incorrect diagnosis of AD. A previous EEG study [29] found no significant differences between DLB patients with and without AD co-pathology. The possibility that subjects in the AD cohort misclassified as DLB were in fact AD-DLB mixed pathology cannot be ruled out. In a future study, we need to systematically utilize both DaTSCAN^®^ and MIBG scintigraphy to select subjects both in the DLB and AD cohorts to enhance the likelihood of the clinical diagnoses being correct.

In this study, the use of AChEIs was allowed due to ethical concerns and to facilitate timely enrollment. Analyses excluding subjects taking cholinesterase inhibitors were done a posteriori, although it should be noted that such a post-hoc analyses can be biased. It is known that cholinergic activity affects EEG. There have been numerous studies of EEG changes following scopolamine administration, and scopolamine has been found to cause background EEG slowing [30]. In AD, cholinergic neurons in the basal forebrain are lost. Deficiencies in cholinergic neurons have been reported to result in changes in the background alpha-wave activity (slowing, decreasing amplitude, decreasing periodic oscillatory changes, and generalization), appearance of slow waves, decreasing fast waves, and irregularities in rhythm formation [31]. The cholinergic deficits are greater and occur earlier in DLB compared to AD [32, 33]. Cholinergic losses in DLB affect both brainstem and basal forebrain presynaptic nuclei, in contrast to AD [34]. In patients with DLB, marked slowing of background activity (lack or slowing of alpha waves), frequent slow wave intrusion, transient slow wave activity in the temporal region, and large fluctuations in background activity have been reported [35, 36]. The greater slowing of the EEG in DLB than in AD is thought to be related to a greater loss of cholinergic neurons in DLB. AChEIs are used to treat the symptoms of DLB and AD based on its neurochemical features [37, 38]. AChEIs affect EEG as well as cognitive functions in DLB and AD. Several studies have shown that AChEIs affect resting state EEG rhythms in AD patients. After a few weeks of the treatment, delta or theta rhythms decrease, dominant alpha rhythms increase, and cognitive functions slightly improve [39, 40]. On the other hand, there is only one cross-sectional study exploring the effects of donepezil on EEG [41] found a significantly lower EEG power density in the delta and theta bands in DLB subjects taking donepezil than in subjects not taking donepezil, whereas there was no significant difference in AD patients. This means that there would be differential effect of AChEIs in DLB and AD. As the cholinergic deficit may partly account for the EEG slowing in DLB and AD, the administration of AChEIs can reverse the EEG slowing in both diseases [42, 43]. However, the loss of cholinergic neurons projecting to the cortex is greater and has a faster progression in DLB compared to AD [44]. Therefore, the use of AChEIs could reduce the value of MC-004 by masking the EEG features of DLB and resulting in patients being misdiagnosed as AD. In fact, in the present study, misclassified subjects in the DLB cohort were somewhat more often taking high doses of donepezil than subjects who were correctly classified. Therefore, use of the MC-004 algorithm should be used prior to the use of high doses of donepezil and avoided in subjects taking high doses of donepezil. For subjects taking galantamine or rivastigmine, which have a short plasma half-life, it would be recommended to ask subjects to postpone taking their daily dose until after EEG recording. Future studies should include only subjects naïve to donepezil, or limit the use of AChEIs as described above, in order to evaluate the true performance of MC-004. This is not an obstacle for using MC-004 as a diagnostic tool, because it would be utilized for patients at an initial stage of diagnosis, where no or only a low dosage of AChEIs has been started.

Moreover, DaTSCAN^®^ /MIBG and AD biomarkers (cerebrospinal fluid or positron emission tomography) are highly desirable in follow-up studies. Longitudinal follow-up for autopsy verification of the patients would also be useful. As most patients with DLB have co-existing AD pathology, the impact of it should be determined in the future studies.

## Conclusion

In conclusion, the qEEG-based machine learning algorithm, MC-004 was able to discriminate between DLB and AD with accuracy of 79.5% (95% CI 63.5–90.7%). MC-004 is a promising biomarker for DLB, and has the potential to improve the detection of DLB in a diagnostic process. Further validation studies based on more proper population, such as those with controlled AChEIs use and with biomarker-supported diagnoses, are indicated.

## Supporting information

S1 Checklist TREND statement checklist. (PDF)

S1 Fig (TIFF)

S1 Appendix Neuropsychological results of individual participants. (DOCX)

S1 File (DOCX)

S2 File (DOCX)

S1 Data (XLSX)
