# Supplementary figures and images for: A prospective multicenter validation study of a machine learning algorithm classifier on quantitative electroencephalogram for differentiating between dementia with Lewy bodies and Alzheimer’s dementia
Source: PLoS One. 2022 Mar 31;17(3):e0265484. doi: 10.1371/journal.pone.0265484 (PMC8970386; doi:10.1371/journal.pone.0265484)

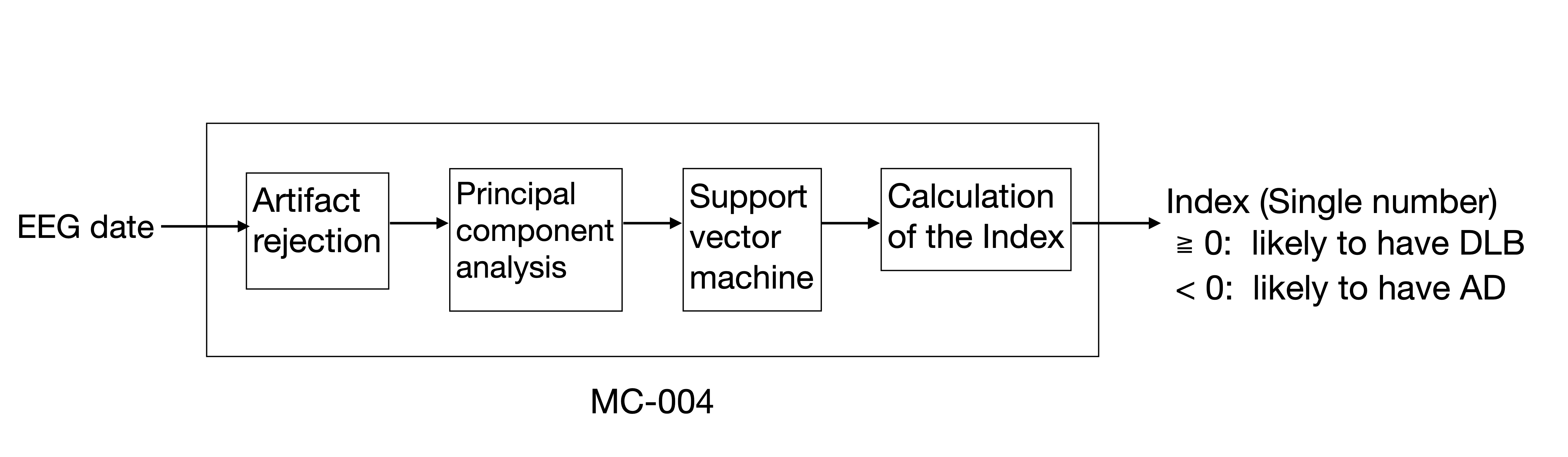

Supplement: S1 Fig — (TIFF) [file pone.0265484.s002.tiff]
