# Supplementary material for: A prospective multicenter validation study of a machine learning algorithm classifier on quantitative electroencephalogram for differentiating between dementia with Lewy bodies and Alzheimer’s dementia
Source: PLoS One. 2022 Mar 31;17(3):e0265484. doi: 10.1371/journal.pone.0265484 (PMC8970386; doi:10.1371/journal.pone.0265484)
Supplement: S1 Appendix — (DOCX) [file pone.0265484.s003.docx]

**実施医療機関及び研究責任者**

大阪大学医学部附属病院　神経科精神科（池田学）

公益財団法人浅香山病院 精神科（釜江和恵）

医療法人花咲会かわさき記念病院（長濱康弘）

独立行政法人国立病院機構仙台西多賀病院 脳神経内科（馬場徹）

東北医科薬科大学病院 脳神経内科（菊池大一）

公益財団法人日本生命済生会日本生命病院 神経科精神科（青木保典）
