# Supplementary material for: A prospective multicenter validation study of a machine learning algorithm classifier on quantitative electroencephalogram for differentiating between dementia with Lewy bodies and Alzheimer’s dementia
Source: PLoS One. 2022 Mar 31;17(3):e0265484. doi: 10.1371/journal.pone.0265484 (PMC8970386; doi:10.1371/journal.pone.0265484)
Supplement: S1 File — (DOCX) [file pone.0265484.s004.docx]

Title:
An investigator-initiated clinical research on the ability of an electroencephalogram analysis program to differentiate between dementia with Lewy bodies (DLB) and Alzheimer's disease (AD)

Research Protocol

Prepared on 11 March 2019, Version 6

1. **Overview**

| Subject | Description |
| --- | --- |
| Objective | The objective of this research is to determine how accurately the electroencephalogram (EEG) analysis program provided by Mentis Cura can identify subjects with probable DLB among subjects including those with probable AD. In a previous study in Norway, when patients with DLB were identified among a subject population including those with AD by using an EEG analysis program, sensitivity was 85% and specificity was 87%. This research is, as a prior step to a large study planned to follow, designed to investigate the feasibility of a large study in Japan and the reproducibility of the result above in a similar Japanese patient population. |
| Primary endpoint | Accuracy of the EEG analysis program in identifying subjects with a diagnosis of probable DLB and subjects with a diagnosis of probable AD (on the basis of the clinical diagnostic criteria) |
| Inclusion criteria | (Inclusion criteria)  Subjects must meet all the following inclusion criteria to qualify for enrollment into the research:   1. 50–90 years of age, inclusive. 2. Patients with DLB, if applicable, who are identified as suffering from probable DLB according to the revised consensus criteria (McKeith et al. 2017). Patients with AD, if applicable, who are identified as suffering from probable AD according to the NIA-AA criteria (McKhann et al. 2011), except for patients with probable AD who meet the criteria for possible DLB (1 core feature only is present, or ≥1 indicative biomarkers only are present). To confirm this, the subject must have undergone sufficient clinical evaluation. 3. Mini-Mental State Examination (MMSE) score 14–26, inclusive. 4. Results available from magnetic resonance imaging (MRI) or computed tomography (CT) scan performed within 12 months prior to the screening visit to rule out possible vascular dementia. 5. Subject or responsible caregiver provides informed consent for the subject to participate in the research.   (Exclusion criteria)  Subjects will be excluded from research participation if one of the following exclusion criteria applies:   1. Subject or responsible caregiver is unable or unwilling to give informed consent. 2. Presence of local or diffuse vascular lesions (e.g., lobar, strategic, or multiple infarcts/hemorrhage, moderate/severe white matter lesions) on MRI or CT, assessed within the current screening visit or within 12 months prior to the screening visit, where this is thought to be the cause of, or contribute to the severity of the subject's dementia. 3. Suffering from a significant neurologic disease other than probable DLB or AD, such as: Parkinson's disease dementia accompanied by dementia (PDD; i.e., subjects with a diagnosis of Parkinson's disease at least 1 year prior to onset of dementia), frontotemporal dementia, Huntington's disease, normal pressure hydrocephalus, brain tumor, progressive supranuclear palsy, seizure disorder, subdural hematoma, multiple sclerosis, or history of evident head trauma followed by persistent neurologic symptoms, or known structural brain abnormalities. Subjects with a history of brain tumor, subdural hematoma, or other clinically significant (in the judgment of the Principal Investigator) space-occupying lesion on neuroimaging will also be excluded. 4. Systemic diseases possibly contributing to the severity of dementia, such as: human immunodeficiency virus (HIV)-associated neurocognitive disorder (e.g., cognitive impairment/dementia), current vitamin B12 deficiency, positive syphilis serology (unless neurosyphilis has been ruled out), and active thyroid dysfunction/hypothyroidism (including abnormally high or low levels of thyroid stimulating hormone). 5. History of alcohol or drug abuse or dependence within the past 2 years (Diagnostic and Statistical Manual of Mental Disorders, 5th edition (DSM V) criteria) 6. History of schizophrenia (DSM V criteria). 7. Any significant systemic illness or unstable medical condition that may affect EEG. 8. Use of specific medications:   a) Centrally active beta-blockers (propranolol), methyldopa and clonidine within 4 weeks prior to screening, narcotics (short-term use of short-acting opioids for pain is allowed if not administered within 24 hours prior to EEG).  b) Use of anti-Parkinsonism medications other than levodopa/carbidopa (Menesit), or levodopa/benserazide (Madopar) within 4 weeks prior to screening, e.g., amantadine, bromocriptine, pergolide, monoamine oxidase type B inhibitors, agonists of the D2 family receptors (ropinirole, pramipexole, rotigotine).  c) Neuroleptics or narcotic analgesics within 4 weeks prior to screening  d) Long-acting benzodiazepines or barbiturates within 4 weeks prior to screening  e) Use of short-acting anxiolytics or sedative hypnotics ≥2 times per week within 4 weeks prior to screening (note: sedative agents must not be used within 72 hours prior to screening and EEG recording).   1. Patients currently using memantine. Cholinesterase inhibitors are permitted if doses are stable for 4 weeks prior to screening. 2. Use of any investigational drugs within 30 days or 5 half-lives, whichever is longer, prior to screening. 3. Subjects who, in the Principal Investigator's opinion, are unlikely to comply with the EEG recording procedure. |
| Research methodology  (treatment schedule, etc.) | 1. Screening of research subjects (Visit 1)   Each investigator will perform screening of subjects who are considered suitable for participating in this research to assess their eligibility for research participation.   1. Explanation and informed consent (Visit 1)   The informed consent procedure for participation in this research will be conducted for research subjects to obtain written informed consent from them.   1. Observation and measurement parameters (other than EEG) (Visit 1 or Visit 2)   Observation and measurements will be performed for the parameters below. For all parameters, results obtained on or after the date of obtaining informed consent or within 3 months prior to the date of obtaining informed consent will be accepted.  Date of birth, gender, years of education, dominant hand, Clinical Dementia Rating (CDR), MMSE, Noise Pareidolia Test, Movement Disorder Society-Unified Parkinson's Disease Rating Scale Part III (MDS-UPDRS Part III), Neuropsychiatric Inventory-12 (NPI-12), Cognitive Fluctuation Inventory (CFI), REM sleep Behavior Disorder Screening Questionnaire (RBDSQ), head MRI, head CT, cerebral blood flow on SPECT, (123)I-meta-iodobenzylguanidine (MIBG) myocardial scintigraphy, dopamine transporter scintigraphy (whether such imaging exams are performed or not, and if performed, the date and the presence or absence of a finding supportive of DLB)   1. EEG testing (Visit 1 or Visit 2)   Standardized EEG recording will be acquired.   1. Analysis of EEG records with the EEG analysis program   EEG records with personal information deleted will be sent to the server of Mentis Cura via the Internet for analysis. |
| Planned number of participants | Planned number of subjects: (1) Our hospital 8 subjects, (2) Overall (as multicenter research) 40 subjects |
| Planned research period | Date of approval by the director of the research site to 31 March 2020 |

1. **Objective and significance of the research**

The objective of this research is to investigate how accurately the electroencephalogram (EEG) analysis program provided by Mentis Cura can identify subjects with probable DLB among subjects including those with probable AD. In a previous study in Norway (Engedal, K. *et al.* *Geriatr. Cogn. Disord.* 2015), when patients with DLB were identified among a subject population including those with AD by using an EEG analysis program, sensitivity was 85% and specificity was 87%. This research is, as a prior step to a large study planned to follow, designed to investigate the feasibility of a large study in Japan and the reproducibility of the result of the previous study in Norway in a similar Japanese patient population.

1. **Background (justification for the scientific rationality of the research)**

Dementia is one of the greatest problems in contemporary societies. It is estimated that there were 4.62 million elderly patients aged 65 years or above with dementia in the early 2010s in Japan. The number of patients with dementia has been increasing, and is estimated to reach approximately 7 million in 2025. The most common form of dementia is AD, accounting for approximately 60% of patients with dementia, followed by vascular dementia and DLB. In both AD and DLB, early and accurate diagnosis and appropriate medication/care are important for both patients and caregivers. Especially for DLB, early diagnosis is ideal because patients, while being likely to respond well to donepezil, need special care due to their hypersensitivity to antipsychotic medications and symptoms specific to DLB such as visual hallucinations and movement disorders. Diagnoses of AD and DLB are made based on clinical symptoms and tests such as neuropsychological tests, imaging exams (MRI, cerebral blood flow scintigraphy, etc.), and blood tests in light of the diagnostic criteria. However, it is often difficult to differentiate between AD and DLB especially during the earlier stages of these diseases.

EEG is a test frequently used in routine clinical practice to monitor the functional state of the brain. It is simple and non-invasive with subjects. EEG has been receiving attention as a biomarker of cognitive dysfunction and may, therefore, be a potentially useful tool for the diagnosis of dementia. Recent studies have suggested that new methods of EEG analysis may provide a way to differentiate subjects with dementia due to DLB from those with AD (Bonanni, L. et al. *J Alzheimers Dis,* 2016, and others). Furthermore, the diagnostic criteria for DLB published in 2017 (McKeith, I. G. et al. *Neurology,* 2017) include "occipital slowing on EEG" as a supportive biomarker.

Engedal et al. conducted a multicenter study in a population in Norway using an EEG analysis program (Engedal, K. et al. *Geriatr Cogn Disord,* 2015), in which a favorable diagnostic performance in differentiating between AD and DLB was demonstrated (sensitivity [SS] = 85%, specificity [SP] = 87%). Earlier and more accurate diagnosis of DLB enabled by an EEG analysis program is expected to reduce burdens on patients and caregivers, and also be of social benefit in reducing the costs of nursing and medical care.

1. **Subjects (research subject selection policy)**

**4.1. Inclusion criteria**

Subjects must meet all the following inclusion criteria to qualify for enrollment into the research:

1. 50–90 years of age, inclusive.
2. Patients with DLB, if applicable, who are identified as suffering from probable DLB according to the revised consensus criteria (McKeith et al. 2017). Patients with AD, if applicable, who are identified as suffering from probable AD according to the NIA-AA criteria (McKhann et al. 2011), except for patients with probable AD who meet the criteria for possible DLB (1 core feature only is present, or ≥1 indicative biomarkers only are present). To confirm this, the subject must have undergone sufficient clinical evaluation.
3. MMSE score 14–26, inclusive.
4. Results available from magnetic resonance imaging (MRI) or computed tomography (CT) scan performed within 12 months prior to the screening visit to rule out possible vascular dementia.
5. Subject or responsible caregiver provides informed consent for the subject to participate in the research.

**4.2. Exclusion criteria**

Subjects will be excluded from research participation if one of the following exclusion criteria applies:

1. Subject or responsible caregiver is unable or unwilling to give informed consent.
2. Presence of local or diffuse vascular lesions (e.g., lobar, strategic, or multiple infarcts/hemorrhage, moderate/severe white matter lesions) on MRI or CT, assessed within the current screening visit or within 12 months prior to the screening visit, where this is thought to be the cause of, or contribute to the severity of the subject's dementia.
3. Significant neurologic disease other than probable DLB or AD, such as: Parkinson's disease dementia accompanied by dementia (PDD; i.e., subjects with a diagnosis of Parkinson's disease at least 1 year prior to onset of dementia), frontotemporal dementia, Huntington's disease, normal pressure hydrocephalus, brain tumor, progressive supranuclear palsy, seizure disorder, subdural hematoma, multiple sclerosis, or history of evident head trauma followed by persistent neurologic symptoms, or known structural brain abnormalities. Subjects with a history of brain tumor, subdural hematoma, or other clinically significant (in the judgment of the Principal Investigator) space-occupying lesion on neuroimaging will also be excluded.
4. Systemic diseases possibly contributing to the severity of dementia, such as: human immunodeficiency virus (HIV)-associated neurocognitive disorder (e.g., cognitive impairment/dementia), current vitamin B12 deficiency, positive syphilis serology (unless neurosyphilis has been ruled out), and active thyroid dysfunction/hypothyroidism (including abnormally high or low levels of thyroid stimulating hormone).
5. History of alcohol or drug abuse or dependence within the past 2 years (5th edition DSM V criteria)
6. History of schizophrenia (DSM V criteria)
7. Any significant systemic illness or unstable medical condition that may affect EEG.
8. Use of specific medications:
9. Centrally active beta-blockers (propranolol), methyldopa and clonidine within 4 weeks prior to screening, narcotics (short-term use of short-acting opioids for pain is allowed if not administered within 24 hours prior to EEG).

b) Use of anti-Parkinsonism medications other than levodopa/carbidopa (Menesit), or levodopa/benserazide (Madopar) within 4 weeks prior to screening, e.g., amantadine, bromocriptine, pergolide, monoamine oxidase type B inhibitors, agonists of the D2 family receptors (ropinirole, pramipexole, rotigotine).

c) Neuroleptics or narcotic analgesics within 4 weeks prior to screening

d) Long-acting benzodiazepines or barbiturates within 4 weeks prior to screening

e) Use of short-acting anxiolytics or sedative hypnotics ≥2 times per week within 4 weeks prior to screening (note: sedative agents must not be used within 72 hours prior to screening and EEG recording).

1. Patients currently using memantine. Cholinesterase inhibitors are permitted if doses are stable for 4 weeks prior to screening.
2. Use of any investigational drugs within 30 days or 5 half-lives, whichever is longer, prior to screening.
3. Subjects who, in the Principal Investigator's opinion, are unlikely to comply with the EEG recording procedure.
4. **Research methodology**

**5.1. Research design**

This research is exploratory research and serves as a stepping stone to prospective research, cross-sectional research, and a validation study.

**5.2. Planned number of research subjects and its rationale**

Planned number of subjects: (1) Our hospital 8 subjects, (2) Overall (as multicenter research) 40 subjects

Rationale for the planned number of subjects:

In the large study planned to follow this research, the diagnostic performance of the EEG analysis program will be analyzed using a standard 2x2 diagnostic test comparison table (subjects with probable DLB classified as positive for the condition and subjects with probable AD classified as negative for the condition). In light of the feasibility of exploratory research in Japan, a total of 40 subjects comprising 20 subjects with probable DLB and 20 subjects with probable AD are planned to be included in the research. With a sample of this size, a diagnostic accuracy of >65% can be detected with 85% power, assuming one-sided alpha level of 5%.

**5.3. Enrollment method, observation/measurement parameters and methods (including schedule)**

**5.3.1. Research subject screening (Visit 1)**

Each investigator will perform screening of subjects who are considered suitable for participating in this research to assess their eligibility for the research participation.

**5.3.2. Explanation and informed consent (Visit 1)**

Sufficient explanations on the nature of this research will be provided to research subjects who have been judged to meet the inclusion criteria and none of the exclusion criteria as a result of the screening process, and written informed consent will be obtained. If a subject is considered to have insufficient ability to provide consent due to the effect of cognitive impairment, written informed consent can be obtained from his/her legally authorized representative.

**5.3.3. Subject enrollment (any point after screening)**

After obtaining written informed consent, each investigator will access the designated URL and enter the previously declared user name to make a tentative enrollment on the website. At the tentative enrollment on the website, the patient number YY-ZZ will be provided. YY represents the first 2 letters of 3 alphabetical letters for site identification, and ZZ represents the numbers assigned in sequential order of enrollment at each site. Each site will prepare a unique list of identification codes (5 letters AAABB; AAA represents 3 alphabetical letters for site identification and BB represents any 2-digit number set for each patient) in order to protect the privacy of patients and enter these codes as patient IDs (for this research) on the website. The prospective enrollment numbers at the time of tentative enrollment will be used as the actual enrollment numbers once patients have been formally registered.

**5.3.4. Observation and measurement parameters (other than EEG) (Visit 1 or Visit 2)**

Observation and measurements will be performed for the parameters below, and results will be entered in the electronic Case Report Forms. For all parameters, results obtained on or after the date of obtaining informed consent or within 3 months prior to the date of obtaining informed consent will be accepted.

**5.3.4.1. Basic patient information**

Date of birth, gender, years of education (total years of education at elementary, junior high, and high schools, university, and graduate school irrespective of old or new system), dominant hand

**5.3.3.2. DLB/AD-related parameters**

Severity of dementia

Clinical Dementia Rating: CDR

Cognitive function test

Mini-Mental State Examination: MMSE

Noise Pareidolia Test

Assessment of parkinsonism

MDS-UPDRS Part III

Assessment with the use of questionnaires administered to the patient's family (including relationship, gender, and age of the informant)

Neuropsychiatric Inventory-12 (NPI-12)

Cognitive Fluctuation Inventory (CFI)

REM sleep Behavior Disorder Screening Questionnaire (RBDSQ)

**5.3.4.3. Imaging-related parameters**

Head MRI, head CT, cerebral blood flow on SPECT: whether performed or not, and if performed, the date

MIBG myocardial scintigraphy, dopamine transporter scintigraphy: whether performed or not, and if performed, the date and the presence or absence of a finding supportive of DLB

**5.3.5. EEG testing (Visit 1 or Visit 2)**

A standardized EEG recording will be acquired after obtaining informed consent. EEG recording requires sufficient duration and quality such that it can be processed for analysis with the EEG analysis program. "Insufficient" quality implies contamination of EEG artefacts that cannot be avoided by the EEG technician making adjustments, e.g., pacemaker-induced artefacts, and muscle artefacts due to uncontrollable facial spasm. The EEG acquisition manual for this research (Appendix 1) specifies the required duration and quality.

**5.3.6. Analysis of EEG records using the EEG analysis program**

An EEG operator or investigator will access the server of Mentis Cura via the Internet through a desktop application to send EEG data for analysis after deleting personal information. This server will return results to the desktop application. Analysis results will be sent back from Mentis Cura.

**5.3.7. Statistical analysis methodology**

**5.3.7.1. Primary endpoints**

Accuracy of the EEG analysis program to identify subjects with a diagnosis of probable DLB and those with a diagnosis of probable AD (diagnoses are based on both the clinical diagnostic criteria and imaging evidence).

**5.3.7.2. Statistical analysis methods**

The primary efficacy analysis will be performed by the lead Principal Investigator on the population of subjects with a successful EEG recording, who are determined by him/her to meet the criteria for probable DLB or AD based on the clinical and imaging findings.

As with the large study planned to follow this research, a standard 2x2 diagnostic test comparison table (subjects with probable DLB classified as positive for the condition and subjects with probable AD classified as negative for the condition) will be used to analyze the diagnostic performance of the EEG analysis program. With a sample of 40 subjects and assuming sensitivity and specificity values of 84%, a diagnostic accuracy of >65% can be detected with 85% power, assuming one-sided alpha level of 5%.

1. **Research period**

Beginning after the approval for the conduct of the research until 31 March 2020

1. **Procedures for obtaining informed consent, etc.**
2. Title of the research and the fact that approval by the director of the research site has been given concerning its conduct
3. Name of the research site and the name of the principal investigator (including names of collaborating research sites and names of the principal investigators of these collaborating research sites)
4. Objective and significance of the research
5. Research method and period (including purpose of the use of specimens or information acquired from the research subject)
6. Reasons why the subject was asked to enroll in the research
7. Discomforts that the research subjects may be exposed to and potential risks and benefits
8. The fact that research subjects, etc. may withdraw their consent at any time even after they have given consent with regard that the research is commenced or continued
9. The fact that refusal or withdrawal of consent by a research subject, etc. with regard that the research is to be commenced or continued does not result in any disadvantage to the research subject, etc.
10. Means whereby information on the research can be made public
11. The fact that research subjects, etc. can request and obtain or read the research protocol and documents concerning the research methods, as well as the procedure to obtain or read such protocols and documents
12. Handling of personal information, etc. (including process of anonymization, when anonymization is conducted, and that anonymously processed information or anonymized personal information will be prepared if applicable)
13. Means for storage and disposal of specimens and information
14. Status of potential research-related conflicts of interest at the research site, such as sources of funding for the research, as well as research-related conflicts of interest of each investigator, etc., such as his/her individual income
15. Response to consultation, etc. made by research subjects, etc. or other individuals concerned
16. Financial expenditure on or remuneration for the research subject, etc.
17. Response related to the provision of healthcare to the research subjects after completion of the research^*1^
18. In the case where any significant finding concerning the subject's health or genetic characteristics which may be inherited by his/her offspring, etc. may be obtained in the course of conducting the research, handling of the research results related to the research subject (including incidental findings)
19. With respect to specimens and information acquired from the research subject, when any of such may be used or provided to other research site(s) for research in the future that is not identified at the time of obtaining consent, a statement to that effect and the details of use assumed at the time of obtaining consent
20. **Procedures, etc. for obtaining informed consent from legally authorized representatives, etc.**
    1. **Policy for the selection of legal representatives, etc. (who must be adults)**

Basically, an individual expected to speak on behalf of and for the benefit of the subject should be selected as the legally authorized representative while taking the subject's family structure, etc. into consideration. The legally authorized representative should be "the subject's spouse, grown child, grown sibling or grandchild, grandparent, a family member living with the subject, or an individual considered equivalent to these relatives."

- 1. **Justification for participation of individuals who require a legally authorized representative, etc.**

Because the target diseases investigated in this research (AD, DLB) are those associated with cognitive impairment, subjects from whom it is considered difficult to obtain effective informed consent can participate as well.

- 1. **Procedures for obtaining informed consent from legally authorized representatives, etc.**

Informed consent will be obtained from legally authorized representatives, etc. in the same manner as the procedures, etc. for obtaining informed consent from the subjects.

1. **Handling of personal information, etc.**

When research-related data are handled, every effort will be made to protect the patient's personal information.

Investigators will assign and use a new subject identification code when providing a case registration form and a case report to a party outside the investigator's site. Any information from which a party outside the site could potentially identify a patient (such as name, address, and phone numbers) will be excluded. To identify a patient when the site receives an inquiry, the research office will use the subject identification code managed by the investigator or the enrollment number issued by the research office.

1. **Discomforts that research subjects may be exposed to and potential risks and benefits, including comprehensive assessment of such discomforts, risks and benefits as well as measures to minimize such discomforts and risks**

Not applicable

1. **Means for storage and disposal of specimens and information (including records related to information used in the research)**

Principal Investigators will take responsibility for storing important documents related to the conduct of the research, etc. (such as copies of application documents, notifications by the hospital director, copies of applications/reports, list of subject identification codes, consent forms, copies of case report forms, etc., and other documents or records required for assuring the reliability of data) in accordance with "Standard Operation Procedures for the Storage of Specimens and Information, etc., Related to Medical Research Involving Human Subjects," keeping them stored appropriately until the date when 10 years have elapsed since publication of results, such as in the form of articles, and destroying these documents thereafter with attention to personal information.

1. **With respect to specimens and information acquired from the research subject, when any of such may be used or provided to other research site(s) for research in the future that is not identified at the time of obtaining consent from the research subject, etc., a statement to that effect and the details of use assumed at the time of obtaining consent**

Clinical information and EEG records obtained in this research may be used for research related to DLB and AD, etc. in the future. In cases where data are used in the future in a way that is outside the scope of explanations given at the time of obtaining informed consent to participate in this research, this information will be notified on the website of our hospital.

1. **Matters to be reported to the director of the research site and procedures for such reports**

Investigators, etc. will report the following in accordance with the rules of the investigator's site.

- Progress of the research

- Serious adverse events

- Protocol deviations

- Protocol modifications

- Reporting of research completion

1. **Status of potential research-related conflicts of interest at the research site, such as the sources of research funding, as well as research-related conflicts of interest of each investigator, etc., such as his/her individual income**

This research will be conducted under a cooperative research contract with Mentis Cura. Analysis of EEG records will be performed by Mentis Cura. Principal Investigators and Sub-Investigators will declare conflicts of interest related to this research for review and approval by the conflict of interest review board of Osaka University, etc.

1. **Means of disclosing research-related information**

A summary of this research will be registered and published on the public database established by the National University Hospital Council of Japan (the University hospital Medical Information Network Clinical Trials Registry; UMIN-CTR) prior to the conduct of the research. Research progress will be updated as appropriate, and research completion will also be reported without delay.

1. **Means to respond to consultation, etc. made by research subjects, etc. or other individuals concerned**

When consultation, etc. is made by a research subject, etc. or another individual concerned, Investigators, etc. of the site handling the research subject will respond in principle. If there is any difficulty in responding, the research representative or research office will be consulted to take action.

1. **Details of financial expenditure on or remuneration for the research subject, etc.**

Expenses for neurological and neuropsychological testing and EEG will be covered by research funding. Subjects will receive transportation expenses as compensation after completing 2 Visits.

1. **Means to respond in cases where a serious adverse event occurs**

Not applicable and omitted

1. **Whether or not compensation will be offered for research-related injuries and details of such compensation**

Not applicable and omitted

1. **Response related to the provision of healthcare to the research subjects after research completion when the research involves the use of any medical techniques outside those usually employed in medical practice**

Treatments covered by general health insurance will be provided to research subjects who have completed the research.

1. **Handling of research results related to the research subject (including incidental findings) in the case where any significant finding concerning the research subject's health or genetic characteristics which may be inherited by his/her offspring, etc. may be obtained in the course of conducting the research**

EEG analysis results obtained in this research are for research-related purposes only. In principle, patients participating in the research will not be informed of analysis results because diagnostic accuracy is not guaranteed. It must be explained and agreed at the time informed consent to participate in the research is obtained that these results will not be disclosed.

1. **Organizational framework and procedures for monitoring and audit**

Not applicable and omitted

1. **Organizational framework for conducting the research**

Research representative

Etsuro Mori

Professor, Endowed Chair of the Department of Behavioral Neurology and Neuropsychiatry, Osaka University United Graduate School of Child Development

Research office

Head of research office: Etsuro Mori
Endowed Chair of the Department of Behavioral Neurology and Neuropsychiatry, Osaka University United Graduate School of Child Development

Responsible analyst

Head of research office: Etsuro Mori
Endowed Chair of the Department of Behavioral Neurology and Neuropsychiatry, Osaka University United Graduate School of Child Development

Data Center

Department of Medical Innovation, Osaka University Hospital

Research sites and Principal Investigators

Department of Neuropsychiatry, Osaka University Hospital (Etsuro Mori, Manabu Ikeda, Maki Suzuki, Yukiko Suzuki, Hiroyuki Watanabe)

Department of Psychiatry, Asakayama General Hospital (Kazue Kamae)

Kawasaki Memorial Hospital (Yasuhiro Nagahama)

Department of Neurology, Sendai Nishitaga National Hospital (Toru Baba)

Department of Psychiatry, Nippon Life Hospital (Etsuro Mori, Manabu Ikeda, and to be determined)

Mentis Cura (Claes Watndal)

1. **Details** **of work to be entrusted and means of supervision over the** **contractor(s) when some of the tasks related to the research are entrusted**

Not applicable and omitted

1. **Approval of research protocol**

Protocols for our site and other sites will be reviewed together by the ethical review board of our site.
