# Supplementary material for: A prospective multicenter validation study of a machine learning algorithm classifier on quantitative electroencephalogram for differentiating between dementia with Lewy bodies and Alzheimer’s dementia
Source: PLoS One. 2022 Mar 31;17(3):e0265484. doi: 10.1371/journal.pone.0265484 (PMC8970386; doi:10.1371/journal.pone.0265484)
Supplement: S2 File — (DOCX) [file pone.0265484.s005.docx]

課題名：
脳波解析プログラムの
Lewy小体型認知症（DLB）とAlzheimer型認知症（AD）判別能に関する医師主導臨床試験

実施計画書

2019年3月11日作成　第6版

1. **概要**

| 項目 | 内容 |
| --- | --- |
| 目的 | 本試験の目的は、Mentis Cura ASにより提供される脳波解析プログラムを用いて、probable ADを含む被験者集団から、probable DLBを有する被験者を検出する際の精度を明らかにすることである。ノルウェーでの先行研究では、脳波解析プログラムを用いてADを含む被験者集団からDLBの患者を検出したところ、感度= 85%、特異度= 87%であった。本試験では、本試験の後に予定している大規模試験の前段階として、日本での大規模試験の実施可能性と、この結果について日本人の同様な患者集団での再現可能性を検討する。 |
| 主要評価項目 | probable DLBと診断された被験者とprobable ADと診断された被験者とを特定する正診率（臨床的診断基準を基準とする）。 |
| 選択基準 | （選択基準）  本試験に組み入れる被験者は、以下のすべての選択基準を満たしている必要がある。   1. 年齢50～90歳（50歳と90歳を含む） 2. DLB患者については、改訂コンセンサス基準（McKeithら、2017）によりProbable DLBと診断された患者。 AD患者については、NIA-AA基準（McKhannら、2011）で、probable ADと診断された患者。ただし、possible DLB（中核的特徴だけが1項目存在する、または、指標的バイオマーカーだけが1項目以上存在する）を満たすprobable AD患者は含めない。これを確認するために十分な臨床評価を受けていること。 3. MMSEスコア14～26（14と26を含む） 4. スクリーニング来院前12カ月以内に行われた磁気共鳴画像検査（MRI）またはコンピュータ断層（CT）スキャンの結果が入手でき、血管性認知症の疑いが除外される。 5. 代諾者及び研究対象者がインフォームド・コンセントを受け、試験に参加することに同意している。   （除外基準）  以下のいずれかの除外基準に該当する場合には、本試験に参加できない。   1. 代諾者及び研究対象者が同意できないか、または同意に前向きではない。 2. MRIまたはCT検査で、局所性またはびまん性の血管病変（例えば、脳葉型、戦略拠点型または多発性の梗塞／出血、中等度／重度の白質病変）が存在し、この病変が、当該被験者の認知症の原因となっているか、または重症度に寄与していると考えられる場合。この検査は、本試験のためのスクリーニング来院時またはスクリーニング来院前12カ月以内に行われていなくてはならない。 3. Probable/possible DLBまたはAD以外の重大な神経疾患を有する。例えば、認知症を伴うパーキンソン病認知症（PDD：すなわち、当該被験者が認知症を発症する1年以上前にパーキンソン病の診断がある）、前頭側頭型認知症、ハンチントン病、正常圧水頭症、脳腫瘍、進行性核上性麻痺、発作性障害、硬膜下血腫、多発性硬化症、明らかな頭部外傷の既往とその後の継続的な神経症状、または既知の脳の構造的異常。神経画像で、脳腫瘍、硬膜下血腫、またはその他の臨床的に重要な（治験責任医師の判断による）占拠性病変の病歴がある場合も、試験には参加できない。 4. 認知症の重症度に寄与する可能性のある全身疾患。例えば、ヒト免疫不全ウイルス（HIV）関連神経認知障害（例えば、認知障害／認知症など）、現在のビタミンB12欠乏症、梅毒血清反応検査陽性（神経梅毒が除外されない場合）、および、活動性の甲状腺障害／甲状腺機能低下（血清中甲状腺刺激ホルモンレベルの異常高値または低値を含む）など。 5. 過去2年間に、アルコールや薬物の乱用または依存の既往（精神疾患の分類と診断の手引、第5版（DSM-V）基準） 6. 統合失調症の既往（DSM-V基準） 7. 脳波に影響を及ぼす可能性のある任意の重要な全身疾患、または不安定な医学的状態。 8. 特定の薬物療法の使用   a）中枢性βブロッカー（プロプラノロール）、スクリーニング前4週間以内のメチルドパとクロニジン、麻薬（鎮痛のための短時間作用型オピオイドの短期間の使用は、脳波前24時間以内でなければ許容される）。  b）スクリーニング前4週間以内の、レボドパ／カルビドパ（メネシット）またはレボドパ／ベンセラジド（マドパー）以外の抗パーキンソン薬の使用。例えば、アマンタジン、ブロモクリプチン、ペルゴリド、MAO-B阻害薬、ゾニサミド、D2ファミリー受容体のアゴニスト（ロピニロール、プラミペキソール、ロチゴチン）など。  c）スクリーニング前4週間以内の抗精神病薬（ドーパミン受容体アンタゴニスト）または麻薬性鎮痛薬  d）スクリーニング前4週間以内の、長時間作用型ベンゾジアゼピンまたはバルビツレート  e）スクリーニング前4週間以内に、短時間作用型抗不安薬又は催眠鎮静剤を1週間に２回以上使用（注：スクリーニングと脳波記録の前72時間以内に鎮静薬を使用してはならない）。   1. メマンチンを現在使用している患者。コリンエステラーゼ阻害薬については、スクリーニングの4週間前からの用量が安定していれば許容される。 2. スクリーニング前4週間以内または半減期の5倍以内（いずれか長い方）の、任意の治験薬の使用。 3. 研究責任医師の判断で、脳波記録手順に従うことができないと考えられる被験者。 |
| 研究方法  （治療スケジュール等） | 1. 試験対象者のスクリーニング（Visit 1）   各担当医師は、本試験への参加が適当と判断される被験者について、試験参加適格性のスクリーニングを行う。   1. 説明と同意の取得（Visit 1）   代諾者および研究対象者に対して、本試験への参加についてインフォームド・コンセントを行い、書面にて同意を得る。   1. 観察・測定項目（脳波検査以外）（Visit 1ないしはVisit 2）   下記の項目について、観察・測定を行う。いずれの項目も、同意取得日以降もしくは同意取得日前3ヶ月以内に取得されたものを採用する。  生年月日、性別、教育年数、利き手、CDR、MMSE、ノイズパレイドリアテスト、MDS-UPDRS PartⅢ、NPI-12、CFI、RBDSQ  頭部MRI、頭部CT、脳血流SPECT、 MIBG心筋シンチグラフィ、ドパミントランスポータシンチグラフィ（画像検査については実施の有無と、実施ありの場合は実施日とDLBを支持する所見の有無）   1. 脳波検査の施行（Visit 1ないしはVisit 2）   　標準化された脳波記録を取得する。   1. 脳波解析プログラムによる脳波記録の解析   　脳波記録は、個人情報削除後、インターネットを通じてMentis Cura ASのサーバーに送信され、解析される。 |
| 予定参加者数 | 予定症例数：①当院　8　例　②全体（多施設の場合）　 40　例 |
| 実施予定期間 | 機関の長の許可日　～　西暦　2020年　3月　31日 |

1. **研究の目的及び意義**

　本試験の目的は、Mentis Cura ASにより提供される脳波解析プログラムを用いて、probable ADを含む被験者集団から、probable DLBを有する被験者を検出する際の精度を検討することである。ノルウェーでの先行研究(Engedal, K. *et al.* *Geriatr. Cogn. Disord.* 2015)では、脳波解析プログラムを用いてADを含む被験者集団からDLB患者を検出したところ、感度= 85%、特異度= 87%であった。本試験では、本試験の後に予定している大規模研究の前段階として、日本での大規模試験の実施可能性と、ノルウェーでの先行研究の結果について日本人の同様の患者集団での再現可能性を検討する。

1. **背景（研究の科学的合理性の根拠）**

　認知症は、現代社会における最も大きな健康問題の1つである。2010年代前半のわが国における65歳以上の高齢者の認知症患者は462万人と推定されている。認知症患者は増加傾向にあり、2025年には、約700万人に達すると推定されている。認知症の病型としては、ADが最も多く、認知症患者の約6割を占める。ついで、血管性認知症、DLBが多い。AD、DLBともに、早期に正確な診断を受け、適切な薬物治療やケアを受けることが、患者と介護者にとって重要である。特に、DLBに関しては、ドネペジルの効果が得られやすい一方で、抗精神病薬への過敏性や、幻視や運動障害などDLBに特有の症状への対応が必要であり、早期の診断が望ましい。ADおよびDLBの診断は、臨床症状、神経心理検査、画像検査（MRI、脳血流シンチグラフィなど）、血液検査などを行い、診断基準に照らし合わせて行う。しかしながら、特に病初期においては、ADとDLBの鑑別は容易でないことがしばしばある。

　脳波は、脳の機能状態をモニタリングするために、日常診療において頻用される検査である。簡便であり、被験者への侵襲もない。脳波は、認知機能障害のバイオマーカーとして注目されており、脳波解析は認知症診断の有用なツールとなる可能性がある。最近の研究では、脳波解析の新しい方法により、DLBによる認知症患者とAD患者とを鑑別する方法が提供される可能性が示唆されている(Bonanni, L. et al. *J Alzheimers Dis,* 2016など)。また、2017年に発表されたDLB診断基準(McKeith, I. G. et al. *Neurology,* 2017) には、支持的バイオマーカーとして「脳波での後頭部徐波化」が含まれている。

　Engedalらは、ノルウェーの集団を対象に脳波解析プログラムを使用して多施設共同研究を実施した(Engedal, K. et al. *Geriatr Cogn Disord,* 2015)。この研究では、ADとDLBの鑑別において、良好な診断性能を示した（感度［SS］= 85%、特異度［SP］= 87%）。脳波解析プログラムにより、DLBをより早期に正確に診断できるようになることは、患者・介護者の負担軽減のみならず、介護・医療にかかるコストの軽減という観点から社会的にも有益であると考える。

1. **対象（研究対象者の選定方針）**

**4.1. 選択基準**

本試験に組み入れる被験者は、以下のすべての選択基準を満たしている必要がある。

1. 年齢50～90歳（50歳と90歳を含む）
2. DLB患者については、改訂コンセンサス基準（McKeithら、2017）によりprobable DLBと診断された患者。AD患者については、NIA-AA基準（McKhannら、2011）で、probable ADと診断された患者。ただし、possible DLB（中核的特徴だけが1項目存在する、または、指標的バイオマーカーだけが1項目以上存在する）を満たすprobable AD患者は含めない。これを確認するために十分な臨床評価を受けていること。
3. MMSEスコア14～26（14と26を含む）
4. スクリーニング来院前12カ月以内に行われた、磁気共鳴画像検査（MRI）またはコンピュータ断層（CT）スキャンの結果が入手でき、血管性認知症の疑いが除外される。
5. 代諾者および研究対象者に対して、本試験への参加についてインフォームド・コンセントを行い、書面にて同意を得る。

**4.2. 除外基準**

以下のいずれかの除外基準に該当する場合には、本試験に参加できない。

1. 代諾者及び研究対象者が同意できないか、または同意に前向きではない。
2. MRIまたはCT検査で、局所性またはびまん性の血管病変（例えば、脳葉型、戦略拠点型、または多発性の梗塞／出血、中等度／重度の白質病変）が存在し、この病変が、当該被験者の認知症の原因となっているか、または重症度に寄与していると考えられる場合。この検査は、本試験のためのスクリーニング来院時またはスクリーニング来院前12カ月以内に行われていなくてはならない。
3. probable DLBまたはAD以外の重大な神経疾患。例えば、認知症を伴うパーキンソン病（PDD：すなわち、当該被験者が認知症を発症する1年以上前にパーキンソン病の診断がある）、前頭側頭型認知症、ハンチントン病、正常圧水頭症、脳腫瘍、進行性核上性麻痺、発作性障害、硬膜下血腫、多発性硬化症、明らかな頭部外傷の既往とその後の継続的な神経症状、または既知の脳の構造的異常。神経画像で、脳腫瘍、硬膜下血腫、またはその他の臨床的に重要な（治験責任医師の判断による）占拠性病変の病歴がある場合も、試験には参加できない。
4. 認知症の重症度に寄与する可能性のある全身疾患。例えば、ヒト免疫不全ウイルス（HIV）関連神経認知障害（例えば、認知障害／認知症など）、現在のビタミンB12欠乏症、梅毒血清反応検査陽性（神経梅毒が除外されない場合）、および、活動性の甲状腺障害／甲状腺機能低下（血清中甲状腺刺激ホルモンレベルの異常高値または低値を含む）など。
5. 過去2年間に、アルコールや薬物の乱用または依存の既往（精神疾患の分類と診断の手引、第5版（DSM-V）基準）
6. 統合失調症の既往（DSM-V基準）
7. 脳波に影響を及ぼす可能性のある任意の重要な全身疾患、または不安定な医学的状態
8. 特定の薬物療法の使用
9. 中枢性βブロッカー（プロプラノロール）、スクリーニング前4週間以内のメチルド

パとクロニジン、麻薬（鎮痛のための短時間作用型オピオイドの短期間の使用は、脳波前24時間以内でなければ許容される）。

b）スクリーニング前4週間以内の、レボドパ／カルビドパ（メネシット）またはレボ

ドパ／ベンセラジド（マドパー）以外の抗パーキンソン薬の使用。例えば、アマン

タジン、ブロモクリプチン、ペルゴリド、MAO-B阻害薬、ゾニサミド、D2ファミリー受容体のアゴニスト（ロピニロール、プラミペキソール、ロチゴチン）など。

c）スクリーニング前4週間以内の抗精神病薬または麻薬性鎮痛薬

d）スクリーニング前4週間以内の、長時間作用型ベンゾジアゼピンまたはバルビツレート

e）スクリーニング前4週間以内に、短時間作用型抗不安薬又は催眠鎮静剤を1週間に
 ２回以上使用（注：スクリーニングと脳波記録の前72時間以内に鎮静薬を使用し
 てはならない）。

1. メマンチンを現在使用している患者。コリンエステラーゼ阻害薬については、スクリーニングの4週間前からの用量が安定していれば許容される。
2. スクリーニング前4週間以内または半減期の5倍以内（いずれか長い方）の、任意の治験薬の使用。
3. 研究責任医師の判断で、脳波記録手順に従うことができないと考えられる被験者。
4. **研究の方法**

**5.1. 研究のデザイン**

本試験は、前向き研究、横断研究、バリデーション研究の前段階としての探索的研究である。

**5.2. 予定研究対象者数及びその設定根拠**

予定症例数：①当院　8　例　②全体（多施設の場合）　40　例

予定症例数の設定根拠：

　本試験の後に予定している大規模試験では、標準の2x2診断検査比較表（probable DLB被験者は条件に照らして陽性、probable AD被験者は陰性）を使用し、脳波解析プログラムの診断性能を解析する。本邦における探索的研究の実現可能性から、probable DLB 20例、probable AD 20例の計40例を予定症例数とする。このサンプルサイズで65%以上の診断精度であることを、片側α＝5%とした時に85%の検出力で検出できる。

**5.3. 登録方法、観察・測定項目とその実施方法（スケジュールも含む）**

|  | Visit 1 | Visit 2 | Visit 2以降の任意の時点 |
| --- | --- | --- | --- |
| 適格性スクリーニング | ○ |  |  |
| 説明と同意の取得 | ○ |  |  |
| 患者基本情報 | ○（Visit 1もしくはVisit 2） (Visit 1の前後3ヶ月以内の情報を採用する) | |  |
| DLB/AD関連項目 | ○（Visit 1もしくはVisit 2） (Visit 1の前後3ヶ月以内の結果を採用する) | |  |
| 画像関連項目 | 任意　　　　　　　　　　　　　　　　　(Visit 1の前後3ヶ月以内の結果を採用する) | |  |
| 脳波検査 | ○（Visit 1もしくはVisit 2） | |  |
| 脳波解析プログラムによる脳波記録の解析 |  |  | ○ |

**5.3.1. 研究対象者のスクリーニング（Visit 1）**

　各担当医師は、本試験への参加が適当と判断される被験者について、試験参加適格性のスクリーニングを行う。

**5.3.2. 説明と同意の取得（Visit 1）**

　スクリーニングの結果、本試験の選択基準を満たし、かつ、除外基準に抵触しないと判断された研究対象者および代諾者に対して、本試験の内容についてインフォームド・コンセントを得る。認知機能障害の影響で、研究対象者に判断能力がない場合は、自ら意向を表することが可能かどうかに関わらず、代諾者のインフォームド・コンセントをもって、本試験への参加意思とする。

　本研究では、有効なインフォームド・コンセントを得ることが困難であると考えられる研究対象者も対象に加わることがありえる。本研究の対象疾患（AD、DLB）は認知機能障害を生じる疾患であることから、有効なインフォームド・コンセントを得ることが困難であると考えられる研究対象者が対象に加わる可能性を考慮しなければ、研究自体の遂行が困難である。

**5.3.3. 症例登録（Visit 1以降の任意の時点）**

　各担当医師は、書面にて同意を得た後、指定されたURLにアクセスし、予め申告したユーザー名を入力してWebサイトで仮登録を行う。Web上での仮登録時の患者番号は、YY-ZZとなる。YYは施設識別用のアルファベット3文字のうちの最初の2文字、ZZは各施設内での登録順に割り振られる数字である。各施設では、患者のプライバシーに配慮して、独自に識別コード（AAABBの5桁：AAAは施設識別用のアルファベット3文字、Bは患者毎に設定した任意の2桁の数字）のリストを作成しておき、これをWebでの患者ID（本試験用）として入力する。なお、仮登録での登録予定番号は、本登録に移行すれば登録番号となる。

**5.3.4. 観察・測定項目（脳波検査以外）（Visit 1ないしはVisit 2）**

　下記の項目について、観察・測定を行い、その結果を電子症例報告書に入力する。いずれの項目も、同意取得日以降もしくは同意取得日前3ヶ月以内に取得されたものを採用する。

**5.3.4.1. 患者基本情報**

生年月日、性別、教育年数（旧制・新制を問わず小中高校、大学、大学院における就学年数の合計）、利き手

**5.3.3.2. DLB/AD関連項目**

認知症重症度

Clinical Dementia Rating: CDR

認知機能検査

Mini-Mental State Examination: MMSE

ノイズパレイドリアテスト

パーキンソニズムの評価

MDS-UPDRS PartⅢ

家族に対する質問紙による評価（情報提供者の続柄、性別、年齢を含む）

Neuropsychiatric Inventory(NPI-12)

Congnitive Fluctuation Inventory(CFI)

RBD(rapid eye movement sleep behavior disordrer) screening questionnaire (RBDSQ)

**5.3.4.3. 画像関連項目**

頭部MRI、頭部CT、脳血流SPECT：実施の有無、実施有りの場合は実施日

MIBG心筋シンチグラフィ、ドパミントランスポータシンチグラフィ：実施の有無、実施ありの場合は実施日とDLBを支持する所見の有無

**5.3.5. 脳波検査の施行（Visit 1ないしはVisit 2）**

　同意の取得後、標準化された脳波記録を取得する。脳波記録は、脳波解析プログラムによる解析のために十分な記録時間と品質を有していることが必要である。「不十分な」品質とは、検査技師による調整では避けられないアーチファクトが混入していることを意味する。例えば、ペースメーカーによるアーチファクトや抑制できない顔面けいれんによる筋肉アーチファクトなどが挙げられる。本試験の脳波取得マニュアル（付録）には、必要となる記録時間と品質について記載されている。

**5.3.6. 脳波解析プログラムによる脳波記録の解析**

　脳波検査担当者あるいは担当医師は、インターネット経由でMentis Cura ASが管理するサーバー（国内に設置）にアクセスし、個人情報（年齢や性別など）を除いた脳波記録（厳密にはそのコピー）を送信する。脳波記録はMentis Cura ASが管理するサーバーで解析される。解析が行われた後の脳波記録はサーバー上から速やかに削除され、Mentis Cura ASが管理するサーバーには、脳波記録は残らない。

**5.3.7. 統計解析の方法**

**5.3.7.1. 主要評価項目**

probable DLBと診断された被験者とprobable ADと診断された被験者とを特定する脳波解析プログラムの正診率（臨床的診断基準と画像所見に基づいた診断を基準とする）。

**5.3.7.2.統計解析の方法**

有効性の主要解析は、研究責任医師が、臨床所見及び画像所見に基づいて、probable DLB又はprobable ADの基準を満たしていることを確認し、脳波記録が問題なく取得されている被験者集団について行う。

本試験の後に予定している大規模試験と同様に、標準の2x2診断検査比較表（probable DLB被験者は条件に照らして陽性、probable AD被験者は陰性）を使用し、脳波解析プログラムの診断性能を解析する。40症例で感度および特異度を84%とすると、65%以上の正診率を、85%の検出力、片側α=5%で検出できる。

※正診率は、本研究では以下のように定義される。

|  | | 臨床的診断基準 | |
| --- | --- | --- | --- |
|  |  | AD | DLB |
| 脳波解析の結果 | AD | a | b |
|  | DLB | c | d |

正診率(accuracy)＝(a+d)/(a+b+c+d)

1. **研究期間**

実施許可後から西暦　2020年3月31日

1. **インフォームド・コンセントを受ける手続等**
2. 研究の名称及び当該研究の実施について研究機関の長の許可を受けている旨
3. 研究機関の名称及び研究責任者の氏名（共同研究機関の名称及び研究責任者の氏名を含む）
4. 研究の目的及び意義
5. 研究の方法（研究対象者から取得された試料・情報の利用目的を含む）及び期間
6. 研究対象者として選定された理由
7. 研究対象者に生じる負担並びに予測されるリスク及び利益
8. 研究が実施又は継続されることに同意した場合であっても随時これを撤回できる旨
9. 研究が実施又は継続されることに同意しないこと又は同意を撤回することによって研究対象者等が不利益な取扱いを受けない旨
10. 研究に関する情報公開の方法
11. 研究対象者等の求めに応じて、研究計画書及び研究の方法に関する資料を入手又は閲覧できる旨並びにその入手又は閲覧の方法
12. 個人情報等の取扱い（匿名化する場合にはその方法、匿名加工情報又は非識別加工情報を作成する場合にはその旨を含む。）
13. 試料・情報の保管及び廃棄の方法
14. 研究の資金源等、研究機関の研究に係る利益相反及び個人の収益等、研究者等の研究に係る利益相反に関する状況
15. 研究対象者等及びその関係者からの相談等への対応
16. 研究対象者等への経済的負担又は謝礼について
17. 研究対象者への研究実施後における医療の提供に関する対応^※1^
18. 研究の実施に伴い、研究対象者の健康、子孫に受け継がれ得る遺伝的特徴等に関する重要な知見が得られる可能性がある場合には、研究対象者に係る研究結果（偶発的所見を含む）の取扱い
19. 研究対象者から取得された試料・情報について、同意を受ける時点では特定されない将来の研究のために用いられる可能性又は他の研究機関に提供する可能性がある場合には、その旨と同意を受ける時点において想定される内容
20. **代諾者等からインフォームド・コンセントを受ける場合の手続**
    1. **代諾者等の選定方針（代諾者等は成人に限る）**

代諾者としては、研究対象者の家族構成等を勘案して、研究対象者の意思および利益を代弁できると考えられる者を選択することを基本とし、「研究対象者の配偶者、成人の子、成人の兄弟姉妹もしくは孫、祖父母、同居の親族又はそれらの親近者に準ずると考えられる者」とする。

- 1. **代諾者等が必要な者の研究参加が必要不可欠な理由**

本試験の対象疾患（AD、DLB）は認知機能障害を生じる疾患であることから、有効なインフォームド・コンセントを得ることが困難であると考えられる研究対象者も参加し得る。

- 1. **代諾者等からインフォームド･コンセントを得る手続き**

研究対象者からインフォームド・コンセントを受ける手続等と同様に、代諾者等からインフォームド・コンセントを得る。

1. **個人情報等の取扱い**

試験に関するデータを取り扱う際は、患者の個人情報保護に最大限の努力を払う。

担当医師は、症例登録票および症例報告書等を当該医療機関外に提供する際には、新たに被験者識別コードを付し、それを用いる。医療機関外のものが患者を特定できる情報（氏名、住所、電話番号など）は記載しない。事務局が医療機関へ照会する際の患者の特定は、担当医師が管理する被験者識別コードまたは事務局が発行した登録No.を用いて行う。

1. **研究対象者に生じる負担並びに予測されるリスク及び利益、これらの総合的評価並びに当該負担及びリスクを最小化する対策**

該当なし

1. **試料・情報（研究に用いられる情報に係る資料を含む。）の保管及び廃棄の方法**

各施設の研究責任者は、研究等の実施に関わる重要な文書（申請書類の控、病院長からの通知文書、各種申請書・報告書の控、被験者識別コードリスト、同意書、症例報告書等の控、その他データの信頼性を保証するのに必要な書類または記録等）については、個人情報に配慮して論文等の成果発表後10年が経過した日まで適切に保存し、その後は個人情報に注意して廃棄する。

1. **研究対象者から取得された試料・情報について、研究対象者等から同意を受ける時点では特定されない将来の研究のために用いられる可能性又は他の研究機関に提供する可能性がある場合には、その旨と同意を受ける時点において想定される内容**

本試験で取得された臨床情報および脳波記録は、将来DLBおよびADに関する試験などに使用される可能性がある。この試験への参加同意取得時の説明の範囲を超えて、将来データを使用する場合は、当院のホームページ内にて告知を行う。

1. **研究機関の長への報告内容及び方法**

研究者等は当該研究機関のルールに則り、以下の報告を行う。

・本研究の進捗状況

・研究計画書からの逸脱

・研究計画書の変更

・研究終了の報告

1. **研究の資金源等、研究機関の研究に係る利益相反及び個人の収益等、研究者等の研究に係る利益相反に関する状況**

本試験は、Mentis Cura ASとの受託研究契約に基づいて行う。また、脳波記録の解析はMentis Cura ASが行う。研究責任者、研究分担者は、本試験に関わる利益相反に関して申告し、各施設の利益相反審査委員会等の審査及び承認を受ける。

1. **研究に関する情報公開の方法**

本研究の概要は、国立大学附属病院長会議が設置している公開データベース（UMIN-CTR）に、本研究の実施に先立って登録され、公開される。また、研究の進捗を適宜更新し、研究の終了についても遅延なく報告する。

1. **研究対象者等及びその関係者からの相談等への対応**

研究対象者等及びその関係者から相談等があった場合は、原則、当該研究対象者の医療機関の研究者等が対応する。対応に苦慮することがある場合には、研究代表者または研究事務局に相談し、措置を講じる。

1. **研究対象者等への経済的負担又は謝礼の内容**

研究対象者に実施する神経学的検査、神経心理検査、脳波検査の費用は研究費にて負担する。研究のための検査目的で来院した場合は、1回のVisitごとに5000円を謝礼として支払う。通常の診療のための来院は、これには含まれない。

1. **重篤な有害事象が発生した際の対応**

該当しないため記載省略

1. **当該研究によって生じた健康被害に対する補償の有無及びその内容**

該当しないため記載省略

1. **通常の診療を超える医療行為を伴う研究の場合の研究対象者への研究実施後における医療の提供に関する対応**

研究対象者への研究実施後においては、通常の保険診療を実施する。

1. **研究の実施に伴い、研究対象者の健康、子孫に受け継がれ得る遺伝的特徴等に関する重要な知見が得られる可能性がある場合の研究対象者に係る研究結果（偶発的所見を含む。）の取扱い**

本研究で行う脳波解析の結果は、あくまでも研究として行う。診断精度が保障されているものではないため、原則、本研究に参加した患者本人に解析の結果は通知しない。研究参加の同意取得の際に「開示しない」旨を説明し、同意を得ておく。

1. **試験全体の中止および中断の基準**

1)研究対象者の安全性もしくは本試験の実施に悪影響を及ぼす可能性のある重大な
　情報を入手した場合
2)実施医療機関が、人を対象とする医学系研究に関する倫理指針もしくは実施計画
 書に違反することにより、適正な試験の実施に支障を及ぼしたと認める場合

3)その他の理由により、研究代表者が、本試験を中断・中止すべきであると判断し
 た場合

1. **研究対象者毎の中止の基準**

1)研究代表者及び研究責任者は、試験の継続が研究対象者に危険を及ぼすおそれが
 判断した場合には、直ちに当該研究対象者について試験を中止する

2)研究代表者及び研究責任者は、研究対象者もしくは代諾者が研究参加の同意を撤

回した場合、直ちに当該研究対象者について試験を中止する

1. **モニタリング及び監査の実施体制及び実施手順**

該当しないため記載省略

1. **研究の実施体制**

研究代表者

大阪大学大学院連合小児発達学研究科行動神経学・神経精神医学寄附講座　教授

森悦朗

研究事務局

大阪大学大学院連合小児発達学研究科行動神経学・神経精神医学寄附講座
事務局責任者：森悦朗

解析責任者

大阪大学大学院医学系研究科内科系臨床医学専攻情報統合医学精神医学　教授

池田学

データセンター

大阪大学医学部附属病院未来医療開発部

実施医療機関及び研究責任者

別添（実施医療機関及び研究責任者）参照

1. **研究に関する業務の一部を委託する場合の業務内容及び委託先の監督方法**

該当しないため記載省略

1. **研究計画書の承認**

大阪大学医学部附属病院倫理審査委員会で承認後、各実施医療機関で審査を行う。
